# DOCK8 gene mutation alters cell subsets, BCR signaling, and cell metabolism in B cells

**DOI:** 10.1038/s41419-024-07180-w

**Published:** 2024-12-01

**Authors:** Heng Gu, Miaomiao Xie, Siyu Zhao, Xi Luo, Yanmei Huang, Lu Yang, Fei Guan, Jiahui Lei, Chaohong Liu

**Affiliations:** 1 Department of Pathogen Biology, School of Basic Medicine, Tongji Medical College and State Key Laboratory for Diagnosis and Treatment of Severe Zoonotic Infectious Diseases, Huazhong University of Science and Technology, Wuhan, China; 2 Department of Anesthesiology, Union Hospital, Tongji Medical College, Huazhong University of Science and Technology, Wuhan, China; 3 Key Laboratory of Anesthesiology and Resuscitation, Ministry of Education, Huazhong University of Science and Technology, Wuhan, China; 4 Health Commission of Henan Province Key Laboratory for Precision Diagnosis and Treatment of Pediatric Tumor, Children’s Hospital Affiliated to Zhengzhou University, Zhengzhou, China

**Keywords:** Lymphocytes, Immunological disorders

## Abstract

DOCK8 deficiency has been shown to affect the migration, function, and survival of immune cells in innate and adaptive immune responses. The immunological mechanisms underlying autosomal recessive (AR) hyper-IgE syndrome (AR-HIES) caused by DOCK8 mutations remain unclear, leading to a lack of specific therapeutic options. In this study, we used CRISPR/Cas9 technology to develop a mouse model with a specific DOCK8 point mutation in exon 45 (c.5846C>A), which is observed in patients with AR-HIES. We then investigated the effect of this mutation on B cell development, cell metabolism, and function in a mouse model with Dock8 gene mutation. The results demonstrated that *Dock8* gene mutation inhibited splenic MZ and GC B cell development and crippled BCR signaling. In addition, it resulted in enhanced glycolysis in B cells. Mechanistically, the reduced BCR signaling was related to decreased B cell spreading, BCR clustering, and signalosomes, mediated by inhibited activation of WASP. Furthermore, the DOCK8 mutation led to increased expression of c-Myc in B cells, which plays an important role in glycolysis. As such, GC B cells’ formation and immune responses were disturbed in LCMV-infected mice. These findings will provide new insights into the immunological pathogenesis of primary immunodeficiency disorder caused by DOCK8 mutation.

## Introduction

Autosomal recessive hyper-IgE syndrome (AR-HIES) is a combined primary immunodeficiency disorder (PID). The majority of individuals with AR-HIES experience loss-of-function mutations in *DOCK8*, which is highly expressed in lymphocytes [1, 2]. Dedicator of Cytokinesis 8 (DOCK8) belongs to the DOCK180 superfamily of guanine-nucleotide exchange factors [3]. Lack of DOCK8, due to *DOCK8* gene mutation, results in a type of combined immunodeficiency that presents with a diverse range of clinical symptoms. These can include recurrent infections, various allergic inflammation conditions, autoimmune disorders, and even susceptibility to tumors [4].

DOCK8 deficiency might facilitate heightened IgE production and weaken T-cell receptor responses due to synapse dysfunction [5]. Our previous work, along with studies from other groups, has demonstrated that DOCK8 deficiency significantly impacts B and T cell functions. DOCK8 mutations have been reported to impair marginal zone (MZ) B cells, hinder B cell persistence in germinal centers, and reduce antibody production [6]. Furthermore, these mutations promote Th17 cell differentiation, which may influence asthma onset [7, 8]. DOCK8 also plays a critical role in actin cytoskeleton reorganization, which is essential for immune cell migration to infection sites. Furthermore, in addition to regulating cell spreading and contraction, actin remodeling also interacts with BCR signaling. Our previous study indicates that the lack of DOCK2 results in reduced levels of both proximal and distal signaling molecules in B cell receptors (BCR), such as phosphorylated CD19 (pCD19), phosphorylated Bruton tyrosine kinase (pBTK) and phosphorylated ribosomal protein S6 (pS6) [9]. As a co-receptor, CD19 becomes phosphorylated following BCR engagement and subsequently recruits PI3K [10]. Although mouse DOCK8 mutation causes a humoral immunodeficiency syndrome, which is very similar to that caused by CD19—particularly in cases where CD19 fails to recruit PI3K [11], DOCK8 has a unique function in BCR-integrin signaling in B cells, setting it apart from the role that DOCK2 plays [6, 12]. Furthermore, it has been proved that different DOCK8 mutation sites lead to various phenotypes [13], thus it is important to investigate the effects of DOCK8 mutations on immune function and their specific molecular mechanism.

Research shows that metabolism is crucial for B cell development, activation, and overall function, as well as for their involvement in the humoral immune response [14]. Interestingly, the DOCK family has been implicated in the phosphatidylinositol 3-kinase (PI3K) signaling in vascular development and disease [15]. In macrophages lacking DOCK2, RAC GTPase activation and reactive oxygen species (ROS) production are reduced during fungal infection [16, 17]. Conditional knockout mice lacking DOCK3 in skeletal muscle (Dock3 mKO) exhibit pronounced hyperglycemia and increased fat accumulation, suggesting a role for DOCK3 in metabolic regulation [18]. Additionally, DOCK5 regulates hepatic insulin function and glucose homeostasis by interacting with Akt and influencing mTOR/S6K1 phosphorylation through its effects on Raptor [19]. A recent research based on metabolomics reports that DOCK8 deficiency patients have perturbations in taurine and dipeptides metabolism, indicating altered antioxidation and cell signaling states [20]. However, relatively little research has been done regarding the effect of DOCK8 deficiency on B-cell metabolism, making this emerging field a fertile ground for exploration and discovery.

C-Myc, a protein highly expressed in primary B cells, plays a central role in regulating mitochondrial metabolism and glycolysis within these cells. Studies have shown that mutations in DOCK8 may influence B cell mitochondrial function and glycolytic activity, potentially through the upregulation of C-Myc [21]. Notably, Myc expression leads to a sustained increase in intracellular Ca^2+^ levels, a requirement for Myc-induced proliferation and differentiation in B cells [22]. This suggests that DOCK8 may regulate B cell metabolism by modulating C-Myc expression, thereby impacting BCR signaling. In pre-malignant B cells, excessive Myc expression can stimulate BCR and PI3K-Akt-mTOR pathways, intensifying downstream signaling when the BCR is engaged [23]. Evidence from T cells indicates that c-Myc regulates a range of metabolic genes that control nutrient and metabolite flux [24]. Unlike the in-depth study of c-Myc in T cells, its role in B cell metabolism remains largely unexplored. Investigating DOCK8’s influence on B cell metabolism may offer fresh insights into the immunological mechanisms underlying primary immunodeficiency disorders (PID).

In this study, we examined how DOCK8 deficiency affects B cell development, function, and metabolic activity in a Dock8-mutant mouse model. Additionally, we explored the impact of DOCK8 mutation on BCR signaling and the reorganization of actin.

## Materials and methods

### Animals

A DOCK8 point mutant mouse model was constructed using CRISPR/Cas9 technology in C57BL/6 background mice. Small guide RNAs targeting exon 45 were designed to generate DOCK8 mutant mice. The first mutation site was a silent mutation (GAG-GAA) and the second was a missense mutation (GCC-GAC), which changed the encoded amino acid from alanine to aspartic acid. The mutant mice were referred to as ‘Mut’ mice, while the control mice, born in the same litter without the missense mutation, were referred to as WT mice. The C57BL/6J mice, including both CD45.2^+^ and CD45.1^+^ variants, were obtained from Charles River. All mice were housed in an SPF facility under controlled conditions: 22 °C and 60–65% humidity. Mice aged 6–8 weeks were selected for analysis, with all animal procedures. All animal procedures received approval from the Institutional Animal Care and Ethics Committee at Tongji Medical College.

### Bone marrow chimeras

To generate bone marrow (BM) chimeric mice, male C57BL/6J (CD45.1^+^) recipient mice, aged 8 weeks, underwent 7 Gy of X-ray irradiation for a duration of 6 min. BM cells isolated from WT (CD45.2^+^) or DOCK8-Mut (CD45.2^+^) mice were combined with those from C57BL/6J (CD45.1^+^) mice at a 1:1 ratio and then intravenously transferred into the recipients (5 × 10^6^ cells per recipient) 4 h post-irradiation. After 6–8 weeks of immune reconstitution, the chimeric mice were sacrificed for further analysis.

### Acute lymphocytic choriomeningitis virus (LCMV) infection

To establish the infection model, 8-week-old wild-type (WT) and DOCK8-mutant (DOCK8-Mut) mice were administered an intraperitoneal inoculation of 2 × 10^5^ plaque-forming units (PFU) of LCMV Armstrong strain in 500 µl DMEM, utilizing an insulin syringe. These mice were sampled on day 8 post-infection. Subsequently, splenic lymphocytes were extracted for flow cytometric analysis, while serum samples were also procured for subsequent studies.

### Cell isolation and purification

Bone marrow (BM) cells were exposed to a red blood cell lysis buffer for 3 min to isolate purified lymphocytes from the BM. To extract peritoneal cells from mice, first, the skin on the abdominal area is incised. Then, 10 ml of sterile PBS is injected into the peritoneal cavity using a syringe, followed by gently agitating the mouse to ensure thorough mixing of the peritoneal cells. Finally, the PBS is collected in an EP tube. To isolate and purify splenic B cells, we first performed Ficoll-Hypaque gradient centrifugation to obtain splenic mononuclear cells. Next, we used a complement-mediated lysis system to eliminate T cells. Finally, we employed adherence-based methods to further remove adherent cells, ultimately collecting the non-adherent B cells.

### Flow cytometry and antibodies

To identify B-cell subsets, BM cells were labeled with a range of antibodies, including BV510-anti-B220 (103247), PE-anti-BP-1 (108307), PE/Cy7-anti-CD24 (101822), FITC-anti-CD127 (135008), APC-anti-CD43 (143208), BV421-anti-IgM (406507), all sourced from BioLegend, along with Percp-7AAD (6084701) from BD Pharmingen. For the staining of peritoneal cells, we utilized APC-anti-CD11b (101226), FITC-anti-CD19 (101506), PE-Cy7-anti-CD5 (100622), PerCP/Cy5.5-anti-IgD (405710), and BV421-anti-IgM, all obtained from BioLegend. Splenic lymphocytes were stained with antibodies, primarily from BioLegend and BD Pharmingen. The antibodies included FITC-anti-CD19, PE-anti-CD23, PE-anti-CXCR4, AF647-anti-GL7 (144606), PerCP/Cy5.5-anti-IgD (405710), APC-anti-CD21 (123412), Percp-7AAD (6084701), BV421-anti-CD86, BV510-anti-B220 (103234), PerCP/Cy5.5-anti-CD45.2 (109838), BV421-anti-IgM (406518), APC/Cy7-anti-CD45.1 (110716), APC-Cy7-anti-CD138 (142521), and BV605-anti-Annexin V. Splenic B cell subsets (B220^high^) were gated as follows: FO B cells (IgM^low^IgD^high^), T1 B cells (IgM^high^IgD^low^), T2 B cells (IgM^high^IgD^high^), MZ B cells (CD21^high^CD23^low^) and GC B cells (GL7^high^CD95^high^). CD138 and B220 was used to distinguish PC (B220^low^CD138^high^) from PBC (B220^high^CD138^high^). Tfh cells were stained with rat anti-mouse CXCR5 primary antibody (551961, BD Biosciences) and biotin-goat anti-rat IgG secondary antibody (112-055-143, Jackson ImmunoResearch). Cells were subsequently treated with DyLight 405 streptavidin, PerCP-anti-CD44, PE/Cy7-anti-PD-1, APC/Cy7-anti-TCR-β, APC-anti-CD4, and PE-anti-ICOS, with antibodies primarily from Biolegend. Tfh cells were defined as CD4^high^CD44^high^CXCR5^high^PD-1^high^ cells. Data acquisition was carried out using a tune NxT flow cytometer from Thermo Fisher and the analysis was performed with FlowJo software developed by TreeStar, USA.

### Isotype switching

Isolated splenic B cells were initially placed into 24-well plates, followed by the introduction of specific stimuli to induce various isotype class switching. To stimulate isotype switching to IgG2b and IgG3, 8 × 10^5^ cells were seeded per well and exposed to LPS at a concentration of 10 μg/ml for 4 days. For IgG1 and IgE switching, 4 × 10^5^ cells were treated with a concentration of 15 μg/ml anti-CD40 and 8 ng/ml IL-4, continuing the treatment for 5 days. IgA switching was induced by seeding 8 × 10^5^ cells per well, followed by treatment with 10 μg/ml LPS and 0.5 ng/ml TGF-beta for 5 days. Cells were transferred to 24-well plates for activation with specific stimuli, thereby initiating the process of class switching to different isotypes.

After collecting cells, a staining process on ice was carried out by adding BV510-anti-B220 along with FVS-700 (Live/Dead Viability Stain) for a 15 min incubation. Subsequent steps involved fixing and permeabilizing cells to allow for intracellular staining with several BD Biosciences antibodies, including IgG3 (553403), IgA (559354), IgE (553415), IgG2b (553395), and IgG1 (550083).

### In vitro proliferation and apoptosis

As previously described [25], 1.8 × 10^6^ purified splenic B cells were labeled in medium with 2.5 μM CTV (Celltrace Violet, C34557, Thermo Fisher) in a 500 μl solution. Following this, 6 × 10^5^ cells were planted in 96-well plates. To stimulate the cells, 3 sets of conditions were applied: LPS (working concentration 5 μg/ml) or CpG (working concentration1.5 μg/ml) in 1640 complete medium for 72 h, and F(ab')2 (working concentration 3 μg/ml), IL-4 (working concentration 5 ng/ml), along with anti-CD40 (working concentration 10 μg/ml) for 96 h. After the collection of cells, BV510-B220 and Percp-7AAD were added to label the cells and detect the BV421-CTV channel. Additionally, 8 × 10^5^ spleen B cells were stained with Annexin V and PI for 5 min to detect apoptosis.

### Western blot analysis

Purified B cells were first incubated with F(ab′)_2_ for 30 min, followed by a 10 min incubation with 20 μg/ml SA (streptavidin). Then, the cells were subjected to an activation process in a 37 °C water bath for different time point. After activation, the cells were treated with RIPA buffer, which included protease inhibitors, to perform cell lysis. Following lysis, the obtained lysates were then analyzed by electrophoresis on a 10% SDS-PAGE, followed by immunoblotting with anti-pY antibody (05-321, Millipore), anti-pSHIP (3941S, Cell Signaling Technology), anti-pSYK (2710, Cell Signaling Technology), anti-pWASP (A300-205A, Bethyl Laboratories), anti-DOCK8(11622-1-AP, Proteintech), anti-pAKT (4060L, Cell Signaling Technology), anti-pS6 (4856S, Cell Signaling Technology), anti-pFOXO1 (9461S, Cell Signaling Technology), anti-pmTOR (5536S, Cell Signaling Technology), anti-pPI3K (4228S, Cell Signaling Technology), anti-PKM2 (4053T, Cell Signaling Technology), anti-c-Myc (13987S, Cell Signaling Technology), anti-HIF-1α (39665, Active Motif), anti-SHIP (2728S, Cell Signaling Technology), anti-SYK(13198S, Cell Signaling Technology), anti-BTK (8547S, Cell Signaling Technology), anti-AKT (9272S,Cell Signaling Technology), anti-PI3K (4292S,Cell Signaling Technology), anti-S6 (2217S,Cell Signaling Technology), anti-FOXO1 (2880S,Cell Signaling Technology), anti-mTOR (2983S,Cell Signaling Technology) and β-actin (60008-1-IG-10, Proteintech). Images were captured using a ChemiDoc™ XRS+ system (Bio-Rad, USA).

### Confocal microscopy (CFm)

In the CFm assay, a suspension of highly purified splenic B cells was applied onto slides coated with polylysine to promote adhesion, followed by a 20-min incubation at 37 °C. Then, the cells were activated using AF-594-conjugated F(ab′)_2_ at 37 °C for 0, 5, 10 and 30 min (115-586-068, Jackson ImmunoResearch). Subsequent steps included washing with PBS, fixing in 4% PFA, and permeabilizing with 0.05% saponin. The cells were then stained with several primary antibodies, including pBTK, pSHIP, pY, pWASP, and AF488-phalloidin. and stained with a panel of antibodies: Finally, fluorescent secondary antibodies—Alexa Fluor 488/405 goat anti-rabbit/mouse IgG (A-31553, A-31556, A-11008), all from Thermo Fisher—were applied for visualization. Fluorescence images were captured via a Nikon confocal microscope. The software NIS-elements AR 5.01 was utilized to evaluate the MFI and Pearson's correlation coefficients, with each dataset derived from a sample of about 50 individual cells.

### Total internal reflection fluorescence microscopy (TIRFm)

In the TIRFm assay, splenic B cells were purified and then stimulated using an antigen-tethered lipid bilayer for time points of 3, 5, and 7 min at 37 °C. Cells were subsequently fixed, permeabilized, and incubated for 30 min at 4 °C with a selection of primary antibodies, including anti-pBTK, anti-pSYK, anti-pY, anti-pSHIP, anti-pWASP, and F-actin. Following this, fluorescent secondary antibodies—Alexa Fluor 488/405 goat anti-rabbit/mouse IgG, all from Thermo Fisher—were applied for visualization. Visualization was accomplished through Nikon TIRFm microscopy, followed by subsequent image processing with the NIS-elements AR 5.01 software to assess both the MFI. Data were collected from 30 individual cells per condition.

### Calcium flux

A preparation of isolated splenic B cells, numbering 2 × 10^6^, was incubated with AF-647-conjugated F(ab')_2_ for 10 min on ice, followed by washing with calcium-free HBSS to remove serum. After diluting calcium-sensitive dye Fluo-4AM with Ca^2+^ free-HBSS, the cells were suspended and added to the chamber coated with polylysine, and incubated for 30 min in a water bath at 37 °C. Subsequently, the free dye was washed off, and the fluorescence baseline of 30 s was measured by Nikon confocal microscopy. Afterwards, preheated Biotin- conjugated F(ab’)_2_ anti-mouse Ig(M + G) was added to stimulate cellular activation, and the ensuing calcium flow was monitored through photography for 5 min. Data were analyzed using the NIS-elements AR 5.01 software.

### Measurement of ECAR and OCR

Utilizing the Seahorse XFe24 Analyzer from Agilent Technologies (XFe24), we quantified cellular metabolic rates, focusing on the oxygen consumption rate (OCR) and the extracellular acidification rate (ECAR), in accordance with the guidelines of the manufacturer. This analysis was applied to spleenic B cells harvested from both wild-type and DOCK8-mutated mice, which had been preconditioned with 5 μg/ml F(ab′)_2_ along with 10 μg/ml anti-CD40 for a duration of 24 h in a 24-well format. The XFe24 24-well microplates were prepared by overnight coating with a poly D-lysine solution at a concentration of 50 μg/ml (C0132, Beyotime) and then 2 × 10^6^ stimulated cells were seeded into the plates. The cartridge plate was hydrated in advance with XF calibrant buffer and incubated at a temperature of 37 °C in a CO_2_-free environment overnight. In the OCR assessment, a series of injections were administered, including oligomycin at 1.5 μM, rotenone at 0.5 μM, FCCP at 1 μM and antimycin A at 1 μM. For the ECAR assessment, oligomycin at 2 μM, 2-DG at 5 mM and glucose at 10 mM were sequentially introduced into the ports A, B, and C, respectively. The Seahorse XF24 software was employed to quantify and graphically represent the OCR and ECAR metrics. We use Seahorse XF24 software to collect and visualize the data for OCR and ECAR.

### Enzyme-linked immunosorbent assay (ELISA)

For the quantitative analysis of Mouse IgE, ELISA was conducted following the protocol of the ELISA Kit (RK00170, Ablconal). Plasma samples from mice were diluted to a ratio of 1:1000 and introduced into antibody-precoated 96-well plates, along with bio-antibody and streptavidin-horseradish peroxidase (HRP), and incubated at a temperature of 37 °C. After an hour, the supernatant was removed, and the wells were rinsed extensively. Chromogen solution was then added, and the plates were left to incubate in the dark for an additional 15 min at 37 °C. Finally, the reaction is terminated by the addition of a stop solution, and the optical density at 450 nm was recorded. Wells without plasma samples served as the control group, and each sample was measured in triplicate.

### Statistical analysis

All data analysis, graphing, and statistics were performed and a two-tailed unpaired Student’s t-test was used (Prism 8.0.2, GraphPad Software). Error bars represent mean ± SEM. Statistical significance was set at *P* < 0.05. Statistical significance differences defined as *P < 0.05, **P < 0.01, ***P < 0.001 and ****P < *0.0001*.

## Results

### DOCK8 mutation inhibits the development of splenic MZ and GC B cells in a cell-intrinsic manner

**Fig. 1:**
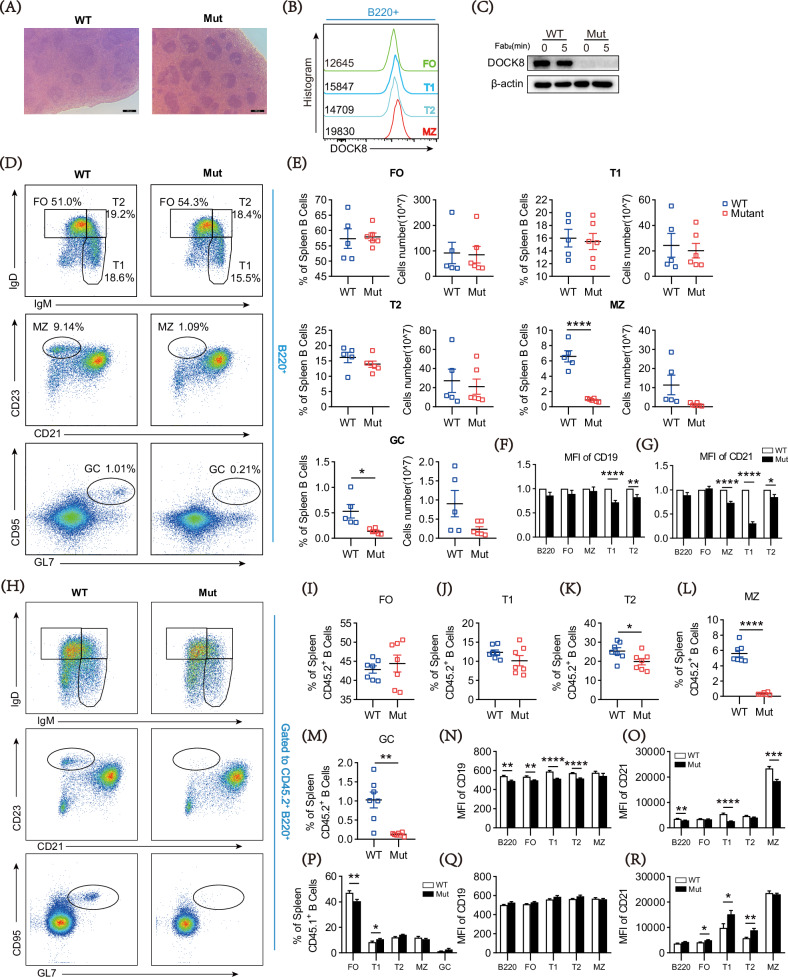
DOCK8 point mutations cause smaller splenic follicles and interfere with peripheral B cell differentiation. **A** H&E staining of spleen sections from 6-week-old WT and DOCK8 mutant mice; scale bars, 200 μm. **B** The expression of DOCK8 in the splenic B cells of WT mice. **C** Immunoblot analysis of DOCK8 expression in B cells from WT and DOCK8 Mut mice stimulated with biotin-conjugated F(ab′)_2_ anti-mouse Ig(M + G) plus streptavidin at 0 and 5 min. **D** Representative dot plots of peripheral B-cell subsets including follicular (FO), transitional type-1 (T1), transitional type-2 (T2), marginal zone (MZ) and germinal center (GC) B cells from WT and DOCK8 Mut mice (n = 5). **E** Quantitative analysis of percentage and cell number of peripheral B-subsets in spleen (n = 5). **F**, **G** The mean fluorescence intensities (MFIs) of CD19 and CD21 subsets of spleen B cells were normalized by DOCK8 Mut/WT ratio. **H** Representative dot plots of peripheral B-cell subsets in CD45.2 WT and DOCK8 Mut chimeras. **I**–**M** Quantitative analysis of FO B, MZ B, GC B, T1 and T2 cell ratios in CD45.2 populations of WT mice and DOCK8 Mut mice (n = 7). **N**, **O** MFIs of CD19 and CD21 in B cell subsets within CD45.2 populations of WT mice and DOCK8 Mut mice (n = 7). Dots represent individual mice and bars denote median in all graphs. **P** Quantitative analysis of FO B, MZ B, GC B, T1 and T2 cell ratios in CD45.1 populations of WT mice and DOCK8 Mut mice (n = 6). **Q**, **R** MFIs of CD19 and CD21 in B cell subsets within CD45.1 populations of WT mice and DOCK8 Mut mice (n = 7). Dots represent individual mice and bars denote median in all graphs. All data are presented as mean ± SEM (*P < 0.05, **P < 0.01, ***P < 0.001, ****P < 0.0001; two-tailed unpaired t-test).

To observe the effect of *Dock8* gene mutation on the physiological phenotype of mice, colon, lung, liver, kidney, and spleen were sectioned and stained with hematoxylin and eosin (H&E). The result indicated that the splenic follicle area of DOCK8 mutation mice was smaller than that of WT mice (Fig. 1A), while other organs were not affected (Supplementary Fig. 1A).

Next, we tested the DOCK8 expression levels of splenic B-cell subgroups in WT mice. It was found that the MZ B cells had the highest expression level relative to follicular (FO), T1, and T2 B cells (Fig. 1B). Interestingly, *Dock8* gene mutation resulted in the deletion of DOCK8 protein in splenic B cells (Fig. 1C). Then we further explored the effects of DOCK8 deficiency on B-cell subsets in the spleen. As Fig. 1D–E depicted, DOCK8 deficiency led to decreased MZ and germinal center (GC) B cells while there was no effect on FO, T1, and T2 B cells. Considering CD19 and CD21 are critical to B cell differentiation [26, 27], expression levels of CD19 and CD21 were assessed in each subgroup of B cells. The results showed that compared with the WT group, the expression of CD19 in T1, and T2 B cell subgroups was significantly decreased (Fig. 1F), and the expression of CD21 in MZ, T1, and T2 subgroups was significantly decreased in the DOCK8 mutation mice (Fig. 1G). Collectively, consistent with the previous report [28], DOCK8 mutation inhibits MZ and GC B cells in the murine spleen.

Given that DOCK8 can affect all cell types in the body, to confirm whether the effects of DOCK8 mutation on MZ and GC B cells were intracellular or context-dependent, we constructed a chimeric mouse bone marrow transplant model, in which irradiated CD45.1 recipient mice were transplanted with mixed CD45.2 bone marrow cells of donor mice (DOCK8 mutation and WT, respectively) with CD45.1 bone marrow cells from recipient mice (Supplementary Fig. 2A). After 8 weeks of immune reconstruction, B-cell phenotypes were observed. Compared with CD45.2^+^ WT B cells, the proportion of MZ and GC B cells in CD45.2^+^ DOCK8 mutation B cells were significantly reduced (Fig. 1H–M), while there was no significant difference in the proportion of MZ and GC B cells between the CD45.1^+^ WT and DOCK8 mutation B cells (Fig. 1P). In addition, CD45.2^+^ DOCK8 mutation B cells had decreased expression of CD19 in total B cells and most of the subpopulations, and inhibited levels of CD21 in total B cells, MZ, and T1 B cells relative to the WT controls (Fig. 1N, O). However, this expression was not observed in CD45.1^+^ mice (Fig. 1Q, R). Taken together, all these results suggest that this inhibition effect of DOCK8 deficiency on MZ and GC cells is cell-intrinsic.

### DOCK8 mutation cripples BCR signaling associated with decreased formation of BCR clusters and signalosomes

**Fig. 2:**
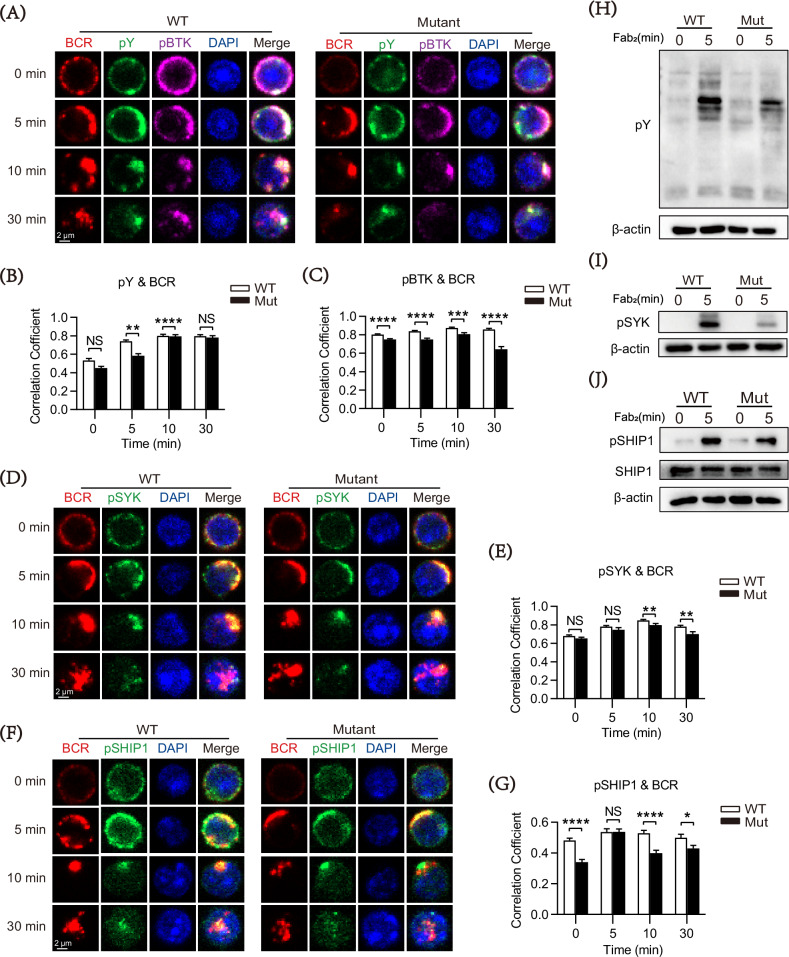
DOCK8 point mutations exhibit reduced proximal BCR signaling. Purified spleen B cells from WT and DOCK8 mutant mice were incubated with AF594-F(ab′)_2_-goat-anti-mouse Ig(M + G) (10 μg/ml) for 30 min at 4 °C and activated for 5, 10, and 30 min at 37 °C, followed by fixation, permeabilization, and staining with markers. **A** Representative confocal microscopy (CFm) images of phosphotyrosine (pY), phosphorylated BTK (pBTK), and BCR (60 × objective, scale bar = 2.5 μm). **B**, **C** Colocalization of pY and pBTK with BCR was analyzed by using NIS-Elements AR 3.2 software. **D** Representative CFm images of phosphorylated SYK (pSYK) and BCR (60×objective, scale bar = 2.5 μm). **E** Colocalization of pSYK with BCR was analyzed by using NIS-Elements AR 3.2 software. **G** Colocalization of pSHIP1 with BCR was analyzed by using NIS-Elements AR 3.2 software. **H, I** Changes in pY and pSYK expression seen for splenic B cells stimulated with biotin-conjugated F(ab′)_2_ anti-mouse Ig(M + G) plus streptavidin for 0 and 5 min by Western blotting. Loading control were total protein and β-Actin. **F** Representative CFm images of phosphorylated SHIP1 and BCR (60 × objective, scale bar = 2.5 μm). **J** Changes in pSHIP1 expression seen for splenic B cells stimulated with biotin-conjugated F(ab′)_2_ anti-mouse Ig(M + G) plus streptavidin for 0 and 5 min by Western blotting. Loading controls were total protein and β-Actin. Data shown in the figures are representative of at least three independent experiments. All data are presented as mean ± SEM (*P < 0.05, **P < 0.01, ***P < 0.001, ****P < 0.0001; two-tailed unpaired t-test).

Since BCR signaling plays an important role in peripheral B cell differentiation and development, we further explored the effect of DOCK8 mutation on BCR signaling with CFm and Western blot. As Fig. 2A revealed, the mutation mice had a lower level of BCRs clustered on B cell surface than the WT group. The colocalization between pY (the total phosphorylated tyrosine signal molecule) and BCR in B cells from the mutation mice was decreased at 5 min and 10 min post sAg stimulation (Fig. 2B). In addition, the coefficient between pBTK (a positive signal molecule in the BCR signaling pathway) and BCR (Fig. 2C) were reduced in the mutation B cells, too. The mutation B cells had lower pSYK levels at 5 min after soluble antigen (sAg) stimulation relative to the WT ones (Fig. 2D) and decreased colocalization between pSYK and BCR at 10 min and 30 min (Fig. 2E). The Western blot results indicated the total levels of BCR signaling (pY) and pSYK of the mutation B cells, were decreased at 5 min post-stimulation (Fig. 2H, I). Furthermore, the colocalization between BCR and pSHIP1 (a negative proximal regulator of BCR) was reduced in the mutated B cells (Fig. 2F, G). Western blot analysis further confirmed that the mutation B cells had a decreased protein level of pSHIP1 in comparison to the WT ones (Fig. 2J). Therefore, DOCK8 mutation down-regulates the activation of BCR proximal signaling, which might be associated with the decreased positive regulating signals pBTK and pSYK.

**Fig. 3:**
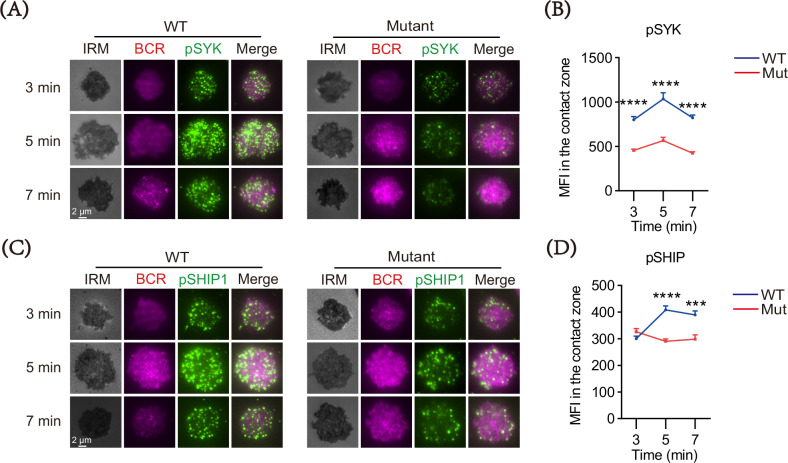
DOCK8 point mutations decrease B cell spreading, BCR cluster formation and BCR signalosome recruitment. Purified splenic B cells from WT and DOCK8 Mut mice were activated with AF647-Fab-goat-anti-mouse Ig (M + G) (10 μg/ml) for 3 min, 5 min, and 7 min, then fixed, permeabilized, and stained for markers following. **A**, **C** Representative total internal reflection fluorescence microscopy (TIRFm) images of pSYK and pSHIP1 (100× objective, scale bar = 5 μm). **B**, **D** MFI of pSYK and pSHIP-1 was performed using NIS-Elements AR 5.0.1 software. Data shown in the figures are representative of at least three independent experiments. All data are presented as mean ± SEM (*P < 0.05, **P < 0.01, ***P < 0.001; two-tailed unpaired t-test).

The previous finding has suggested that the cytoskeleton is closely involved in BCR signaling [29]. Thus, we investigated the impact of DOCK8 mutation on the activation of B cells including BCR clustering and signalosome accumulation. TIRFm was used to observe the formation of BCR clusters and signalosome accumulation in the contact area of splenic B cell interaction with membrane antigen (mAg) tethered to lipid bilayers. As shown in Fig. 3A–D, the accumulation of pSYK and pSHIP1 in the contact area of the mutation B cells was significantly reduced at various time points post mAg stimulation compared with the WT B cells (Fig. 3A–D). Altogether, these data indicate that DOCK8 mutation suppresses BCR cluster formation, and signalosome recruitment during early B cell activation, thereby downregulating proximal BCR signaling.

### DOCK8 mutation decreases the polymerization of actin by pWASP during BCR activation

**Fig. 4:**
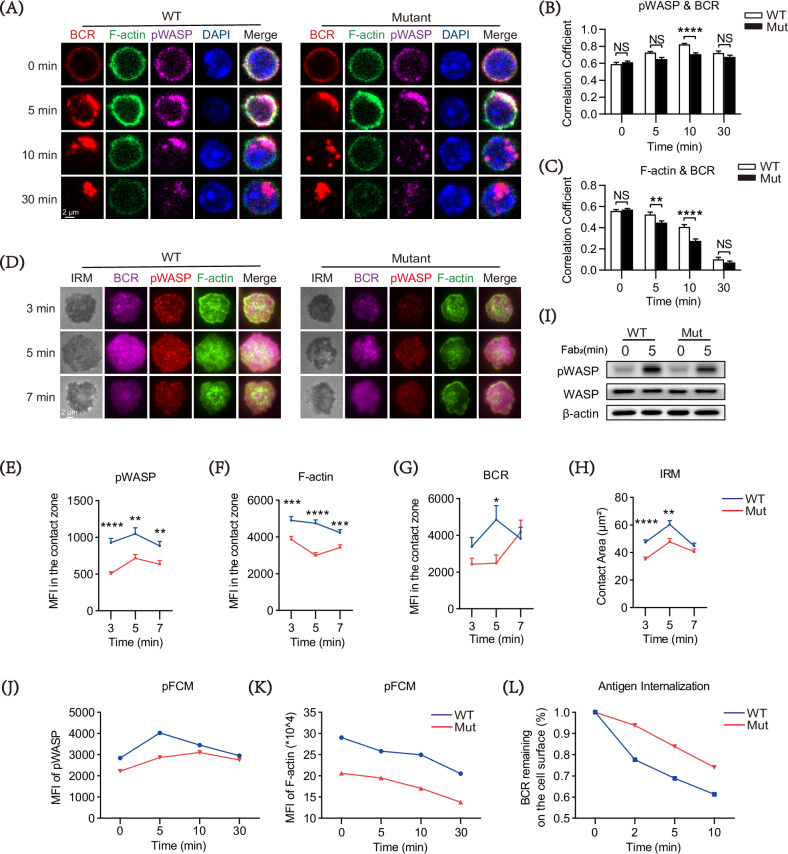
DOCK8 point mutations downregulate the actin polymerization via inhibiting the activation of WASP during BCR activation. **A** Representative confocal microscopy (CFm) images of phosphorylated WASP (pWASP), F-actin, and BCR (60× objective, scale bar = 2.5 μm). **B**, **C** Colocalization of pWASP and F-actin with BCR was performed using NIS-Elements AR 5.0.1 software. **D** Representative total internal reflection fluorescence microscopy (TIRFm) images of pWASP and F-actin at 3 min, 5 min, and 7 min of activation (100× objective, scale bar = 5 μm). **E, F** Quantification of MFI for pWASP and F-actin was performed using NIS-Elements AR 5.0.1 software. **G, H** Quantification of interaction between B cells and mAg in the contact zone, MFI of BCR was performed using NIS-Elements AR 5.0.1 software. **J, K** Splenic B cells were pre-incubated with B220 and then stimulated with sAg for 0, 5, 10, and 30 min. After fixation and permeabilization, cells were stained with pWASP and F-actin and then detected by flow cytometry. The MFI of pWASP and F-actin in B220^**+**^ cells was quantified by FlowJo 10.8.1 software. **L** The ability of B cells to internalize antigens in WT and Mut mice. **I** Changes in pWASP expression seen for splenic B cells stimulated with biotin-conjugated F(ab′)_2_ anti-mouse Ig(M + G) plus streptavidin for 0 and 5 min by Western blotting. Loading control was total protein and β-Actin. Data shown in the figures are representative of at least three independent experiments. All data are presented as mean ± SEM (*P < 0.05, **P < 0.01, ***P < 0.001, ****P < 0.0001; two-tailed unpaired t-test).

Actin depolymerization and repolymerization are regulated by BCR signaling and Wiskott-Aldrich syndrome protein (WASP), and are closely associated with the spread of B cells and the formation of BCR clusters during cell activation [30]. To explore the potential mechanism by which DOCK8 mutation inhibited B cell actin recombination, CFm was used to detect pWASP and actin levels in B cells treated by sAg at various time points. We found that pWASP colocalization with BCR at 10 min were reduced in the DOCK8 mutation B cells compared with the WT ones (Fig. 4A, B). In addition, the mutation B cells had decreased colocalization between F-actin and BCR than the WT B cells post sAg stimulation (Fig. 4A, C). Considering that TIRFm has a higher resolution than CFm, providing better observation of early activation events of B cells, we used TIRFm to verify the above results. Consistent with the findings obtained from CFm, levels of pWASP, F-actin, and BCR in the contact area of the mutation B cells were significantly inhibited (Fig. 4D-G). Interference Reflection Microscopy (IRM) was used to detect the surface area of interaction between B cells and mAg to reflect the expansion of B cells. As shown in Fig. 4H, all B cells spread rapidly with a peak at 5 min post mAg stimulation, and DOCK8 mutation suppresses B cell spreading. The detection of phosphorylated proteins using flow cytometry were consistent with the inhibition effect of DOCK mutation on pWASP and F-actin in activated B cells (Fig. 4J, K). In addition, Western blot analysis revealed a decreased protein level of pWASP in the activated mutation B cells, although no difference in total WASP protein level relative to the control group (Fig. 4I). The normal deformation of the B cell cytoskeleton is the structural basis for maintaining the internalization of antigens by B cells. Consequently, we also assessed the ability of B cells to internalize antigens and found that the DOCK8 mutation impaired this capacity (Fig. 4L). Taken together, these results suggest that DOCK8 mutation disrupts pWASP and actin polymerization, which is associated with the formation of BCR clusters and antigen internalization during B cell activation.

### DOCK8 mutation reprograms cell metabolism and enhances glycolysis in B cells

**Fig. 5:**
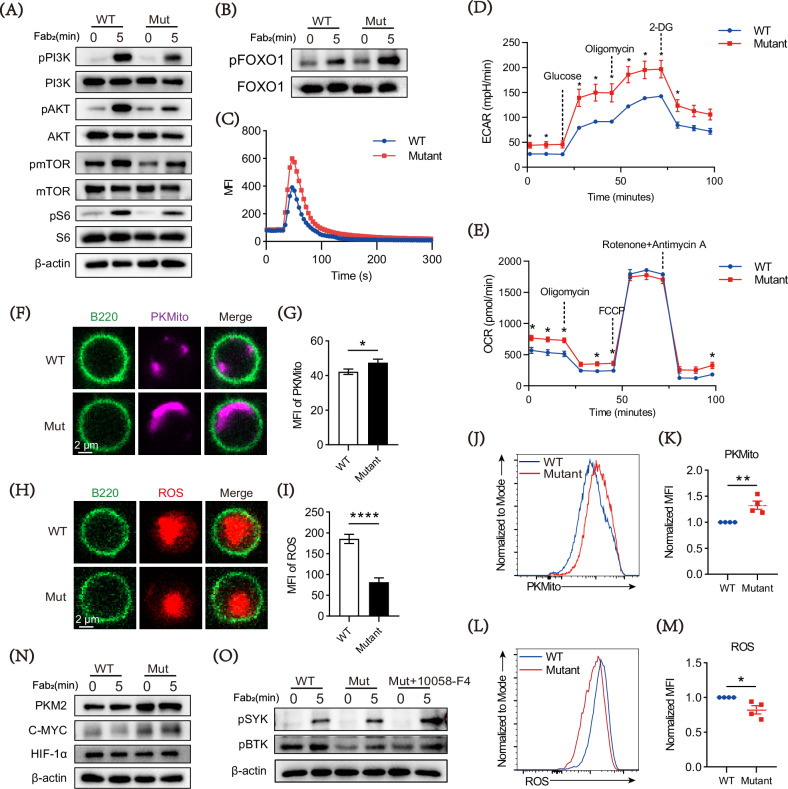
DOCK8 point mutations enhance energy metabolism in B cells. **A**, **B** Changes in pPI3K, pAKT, pmTOR, pS6 and pFOXO1 expression seen for splenic B cells stimulated with biotin-conjugated F(ab′)_2_ anti-mouse Ig(M + G) plus streptavidin for 0 and 5 min by Western blotting. Loading control was total protein or β-Actin. **C** Representative image of intracellular Ca2^+^ flux kinetics measured over 5 min after stimulation of WT and DOCK8 Mut B cells by 10 μg/ml biotin-conjugated F(ab′)2 anti-mouse Ig (M + G). All images are representative of three independent experiments. **D**, **E** Purified splenic B cells from WT and DOCK8 Mut mice were stimulated with F(ab′)2 anti-mouse Ig (M + G) (5 μg/ml), anti-CD40 (10 μg/ml) and IL-4 (5 ng/ml) for 24 h and the ECAR and OCAR were determined using a Seahorse Bioflux analyzer. **F** Representative images of intracellular PKMITO levels after F(ab′)2 anti-mouse Ig(M + G), anti-CD40 plus IL-4 stimulation for 24 h. (60× objective, scale bar = 2.5 μm). **G** MFI of PKMITO was quantified using NIS-Elements AR 5.0.1 software. **H** Representative images of intracellular reactive oxygen species (ROS) levels after F(ab′)2 anti-mouse Ig (M + G), anti-CD40 plus IL-4 stimulation for 24 h. (60× objective, scale bar = 2.5 μm). **I** MFI of ROS was quantified using NIS-Elements AR 5.0.1 software. **J** Representative histogram of intracellular PKMITO levels in B220^+^ cells after F(ab′)2 anti-mouse Ig (M + G), anti-CD40 plus IL-4 stimulation for 24 h were detected by flow cytometry. **K** MFI of PKMITO was quantified using FlowJo 10.8.1 software. **L** Representative histogram of intracellular ROS levels in B220^+^ cells after F(ab′)2 anti-mouse Ig (M + G), anti-CD40 plus IL-4 stimulation for 24 h was detected by flow cytometry. **M** MFI of ROS was quantified using FlowJo 10.8.1 software. **N** Changes in pSYK, pBTK expression seen for splenic B cells stimulated with biotin-conjugated F(ab′)_2_ anti-mouse Ig(M + G) plus streptavidin for 0 and 5 min by Western blotting. Loading control was β-Actin. **O** Changes in pSYK, pBTK expression seen for WT, Mut, and c-Myc inhibitor -treated Mut mouse splenic B cells stimulated with biotin-conjugated F(ab′)_2_ anti-mouse Ig(M + G) plus streptavidin for 0 and 5 min by Western blotting. Loading control was β-Actin. Data shown in the figures are representative of at least three independent experiments. All data are presented as mean ± SEM (*P < 0.05, **P < 0.01, ***P < 0.001; two-tailed unpaired t-test).

Compelling discoveries have shed light on the core of metabolic pathways in determining the cellular fate and function of B cells, although metabolic control of B-cell fate is only partially understood [31, 32]. Since the PI3K signaling is essential for B cell homeostasis [33, 34], next we investigated whether DOCK8 mutation affected the PI3K signaling pathway in B cells. The results showed that the mutation B cells had lower protein levels of pPI3K, pAKT, pmTOR, pS6, and higher pFOXO1 expression than the WT ones (Fig. 5A, B). In addition, calcium flow in the mutation B cells was increased significantly (Fig. 5C).

The PI3K-AKT-mTOR pathway is crucial in mitochondrial biogenesis and involved in glucose metabolism [35, 36]. Therefore, extracellular acylation rate (ECAR) and oxygen consumption rate (OCR) were measured by Seahorse. We found that the mutation B cells had an enhanced basal and maximum glycolysis rate than the WT B cells (Fig. 5D). The basal respiratory rate of the mutated B cell mitochondria was increased, while the maximum aerobic respiratory capacity of the mutated B cells did not differ from that of the WT B cells (Fig. 5E). In addition, we also detected the mitochondrial activity of B cells and the results showed that the mutation B cells had a higher mean fluorescence intensity (MFI) of the mitochondrial mass than the WT B cells under LPS stimulation (Fig. 5F, G, J, K). Furthermore, ROS levels of the mutation B cells were reduced significantly after stimulation (Fig. 5H, I, L, M). All these data suggested that DOCK8 mutation leads to metabolic reprogramming in splenic B cells.

Based on the results of DOCK8 mutation affecting glycolysis and mitochondrial metabolism, and c-Myc and HIF-1α are key molecules regulating mitochondrial metabolism and glycolysis [21, 37], so we detected the protein levels of c-Myc, HIF-1α, and key glycolytic enzyme PKM2. The data showed increased levels of c-Myc and PKM2 in mutant B cells, while no change in HIF-1α protein expression (Fig. 5N). The homeostasis of mitochondrial metabolic functions in B cells is essential for their survival, as well as for supporting their proliferation, activation, and antibody production [34]. Reports have indicated a bidirectional regulation between cellular metabolism and BCR signaling [38]. To further explore if the metabolic dysfunction resulting from DOCK8 deficiency could reciprocally influence BCR signaling, mutant mice B cells were treated with a c-Myc inhibitor. The results showed that this treatment effectively reversed the diminished proximal BCR signaling caused by the DOCK8 mutation (Fig. 5O). Altogether, DOCK8 mutation leads to B cell metabolic reprogramming, manifested primarily in enhanced glycolysis, and it may regulate cellular metabolism through up-regulation of c-Myc, thereby providing feedback regulation of BCR signaling.

### DOCK8 mutation interferes with the formation and immune responses of GC B cells in LCMV-infected mice and alters the isotype class switch of B cells

**Fig. 6:**
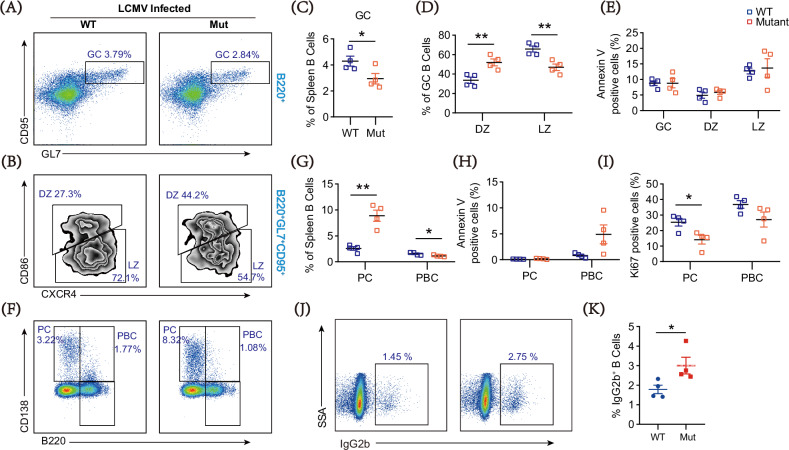
DOCK8 point mutations decrease the proportion of GC B cells and alter the light zone/dark zone ratio. **A** Representative dot plots of germinal center (GC) B cells from WT and DOCK8 Mut mice after LCMV Armstrong infection for 8 days by flow cytometry (n = 4). **B** Representative contour plots of dark zone (DZ) and light zone (LZ) B cells of GC B cells from infected WT and DOCK8 Mut mice by flow cytometry (n = 4). **C**, **D** Quantitative analysis of the proportion of DZ and LZ B cells in GC B cells from infected WT and DOCK8 Mut mice (n = 4). **E** Percentage of Annexin V positive cells in total splenic GC B cells and DZ, LZ B subsets in WT and Mut mice. **F** Representative dot plots of plasma cells (PC) and plasma blast cells (PBC) from infected WT and DOCK8 Mut mice by flow cytometry (n = 4). **G** Quantitative analysis of the proportion of PC and PBC cells in splenic B cells from infected WT and DOCK8 Mut mice (n = 4). **H**, **I** Quantitative analysis of the proportion of Annexin V and Ki67 positive cells in PC and PBC cells from infected WT and DOCK8 Mut mice (n = 4). **J** Representative dot plots for antigen class switching to IgG2b after splenic B cells were stimulated with LPS to induce IgG2b and IgG3 switching. **K** Quantitative analysis of the proportion of IgG2b^+^ cells in splenic B cells from WT and DOCK8 Mut mice (n = 4). Dots represent individual mice and bars denote median in all graphs. All data are presented as mean ± SEM (*P < 0.05, **P < 0.01, ***P < 0.001, ****P < 0.0001; two-tailed unpaired t-test).

Given that DOCK8 mutation affected the development of GC cells and downregulated the activation of the PI3K signaling pathway in B cells, which plays a crucial role in GC response [39], we wondered whether DOCK8 mutation affected GC responses. Acute lymphocytic choriomeningitis virus (LCMV) infection is one of the ideal models for the study of GC immune response and antibody production [40, 41]. In this study, mice were infected with the LCMV- Armstrong virus and sacrificed on Day 8 after infection. We found that compared with those of WT mice, in line with the previous finding (Fig. 1E, F), the proportion of splenic GC cells in DOCK8 mutant mice was reduced (Fig. 6A, C). In addition, the proportion of light zone (LZ) was decreased, and that of dark zone (DZ) was increased in GC B cells of DOCK8 mutant mice (Fig. 6B, D), and no significant differences were observed in the levels of apoptosis in GC B cells and their subpopulations (Fig. 6E). The results indicated that the DOCK8 mutation affected the distribution of GC B cells.

Also, we measured the proportions of plasma cells and plasmablasts (PBC) in splenic GC. The results suggested that DOCK8 mutation mice had a higher proportion of plasma cells and a lower proportion of PBC than the WT (Fig. 6F, G), which might be related to decreased apoptosis of plasma cells (Fig. 6H, I).

Since immunoglobulin class switching in B cells primarily occurs within GC, to further investigate whether the DOCK8 mutation affects the class-switching ability of B cells, we stimulated the cells using LPS, a TLR agonist, in combination with specific cytokines. This approach was chosen to mimic the physiological activation signals required for CSR. The results revealed that compared to WT mice, DOCK8 mutant mice exhibited an increase in IgG2b class switching (Fig. 6J, K), while there were no significant differences in IgA, IgE, IgG1, and IgG3 class switching (Supplementary Fig. 3A–E). Additionally, there were no notable differences in the proliferation and apoptosis levels of B cells between WT and mutant mice (Supplementary Fig. 4A–E). These findings suggest that the DOCK8 mutation interferes with the formation and immune response of GC B cells in LCMV-infected mice and promotes class switching towards IgG2b.

## Discussion

In this study, we demonstrated that *Dock8* gene mutation results in decreased splenic MZ and GC B cell development and crippled BCR signaling, along with enhanced glycolysis. Mechanistically, the reduced BCR signaling might be related to decreased B cell spreading, BCR clustering, and signalosomes, mediated by inhibited activation of WASP. Furthermore, the DOCK8 mutation enhanced the expression of c-Myc in B cells, which plays an important role in glycolysis. As such, GC B cells’ formation and immune responses are disturbed in LCMV-infected mice. These findings provide new insights into the immunological pathogenesis of PID caused by DOCK8 mutation.

Our findings further underscore the cell-intrinsic role of DOCK8 in regulating B cell development, which is consistent with and extends prior research on its significance in MZ and GC B cell maturation [2, 6, 28]. Previous studies have shown that MZ B cell formation depends heavily on BCR-CD19-PI3K signaling [42, 43]. Additionally, Jabara et al. finds that DOCK8 functions as an adaptor that links Toll-like receptor (TLR)-MyD88 signaling to B cell activation [44]. Furthermore, DOCK8 has been implicated in cytoskeletal remodeling, which is important for B cell development and migration [45]. In this study, we detected BCR signaling with CFm and Western blot, along with the formation of BCR clusters and signalosome accumulation with TIRFm. We found that DOCK8 mutation suppresses B cell spreading, BCR cluster formation, and signalosome recruitment during early B cell activation, thereby downregulating proximal BCR signaling. In addition, DOCK8 mutation disrupted pWASP and actin polymerization, which is associated with the formation of BCR clusters during B cell activation. Collectively, DOCK8 mutation leads to impaired development of MZ and GC B cells, and potential mechanisms by which DOCK8 regulates B cells include its involvement in BCR signaling and cytoskeletal remodeling. The study thus provides a deeper understanding of how DOCK8 mutation alters B cell development and immune responses, shedding light on its pathophysiological mechanisms in primary immunodeficiency disorders.

B cells undergo a series of complex metabolic processes to ensure efficient energy production for these vital activities including cell development, antibody production, proliferation, and differentiation. In this study, we found that DOCK8 mutation led to significant metabolic reprogramming in B cells, characterized by enhanced glycolysis and reduced PI3K-AKT-mTOR signaling, which resulted in impaired energy production as shown by disrupted ECAR and OCR. Importantly, we observed upregulation of the c-Myc transcription factor, known to regulate key metabolic pathways, including glycolysis and oxidative phosphorylation. We also found that treating B cells from mutant mice with a c-Myc inhibitor effectively restored the impaired proximal BCR signaling caused by the DOCK8 mutation. This suggests that DOCK8 mutation may alter B cell metabolism through c-Myc upregulation, which could feedback to affect BCR signaling and immune responses. Previous research has demonstrated that c-Myc-deficient B cells experience disrupted glucose metabolism, reduced ATP production, and mitochondrial dysfunction. The study highlighted the crucial role of c-Myc in coordinating B cell metabolism to support their functions [46]. Gaining a deeper understanding of how the c-Myc pathway governs glucose utilization and B cell metabolic processes could offer valuable insights for developing future therapeutic strategies targeting B cell-related diseases.

GC cells and the PI3K signaling play crucial roles in the immune response against viruses [47]. Activation of the PI3K pathway in GC cells has been shown to promote their survival, proliferation, and differentiation, while inhibition of PI3K impairs the survival and expansion of GC cells, leading to reduced antibody production [48]. Our results depicted that DOCK8 mutation led to altered phenotype, and compromised GC immune responses in the LCMV-infected mice, which might be associated with the disturbed metabolic programming and the PI3K pathway, along with crippled BCR signaling.

Based on our findings, DOCK8 mutation disrupts BCR signaling and cytoskeletal remodeling, leading to impaired MZ and GC B cell development, and also induces metabolic reprogramming characterized by enhanced glycolysis and reduced PI3K-AKT-mTOR signaling, thereby affecting immune responses and offering new insights into the pathogenesis of DOCK8-related immunodeficiencies.

## Supplementary information

Supplemental Information

CDDIS-24-3717R-original data-WB

## Data Availability

The data sets and any other raw data that support the findings of this study are available from the corresponding author upon reasonable request.
