# Supplementary figures and images for: DOCK8 gene mutation alters cell subsets, BCR signaling, and cell metabolism in B cells
Source: Cell Death Dis. 2024 Dec 1;15(11):871. doi: 10.1038/s41419-024-07180-w (PMC11608328; doi:10.1038/s41419-024-07180-w)

(A)

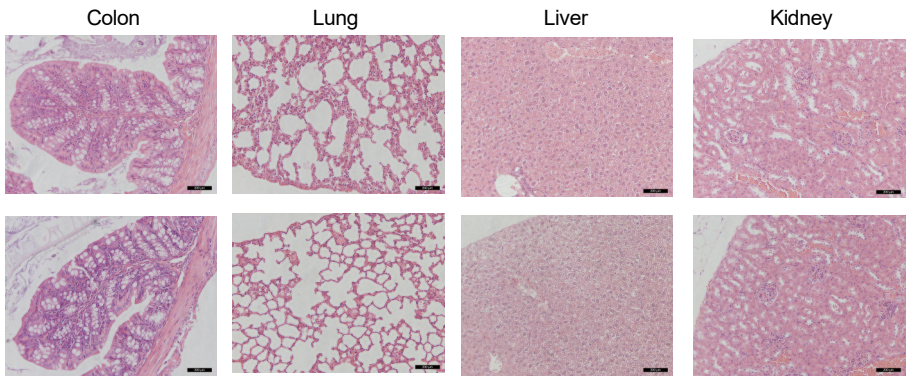

(A)

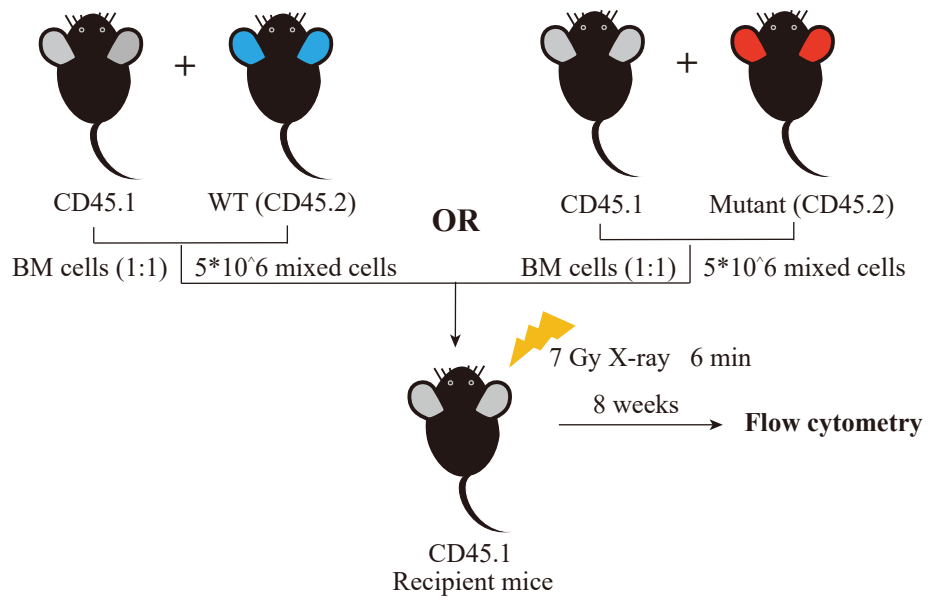

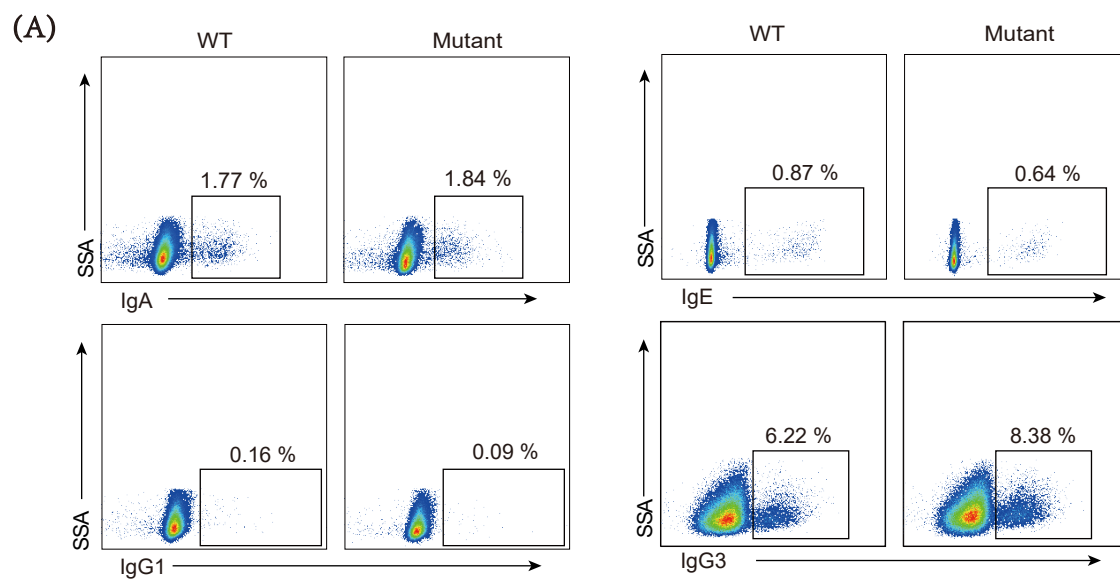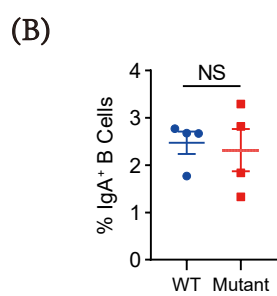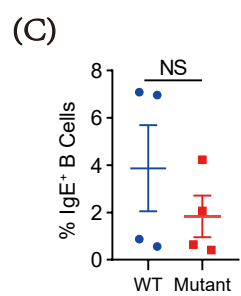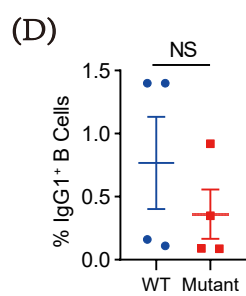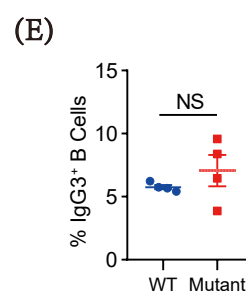

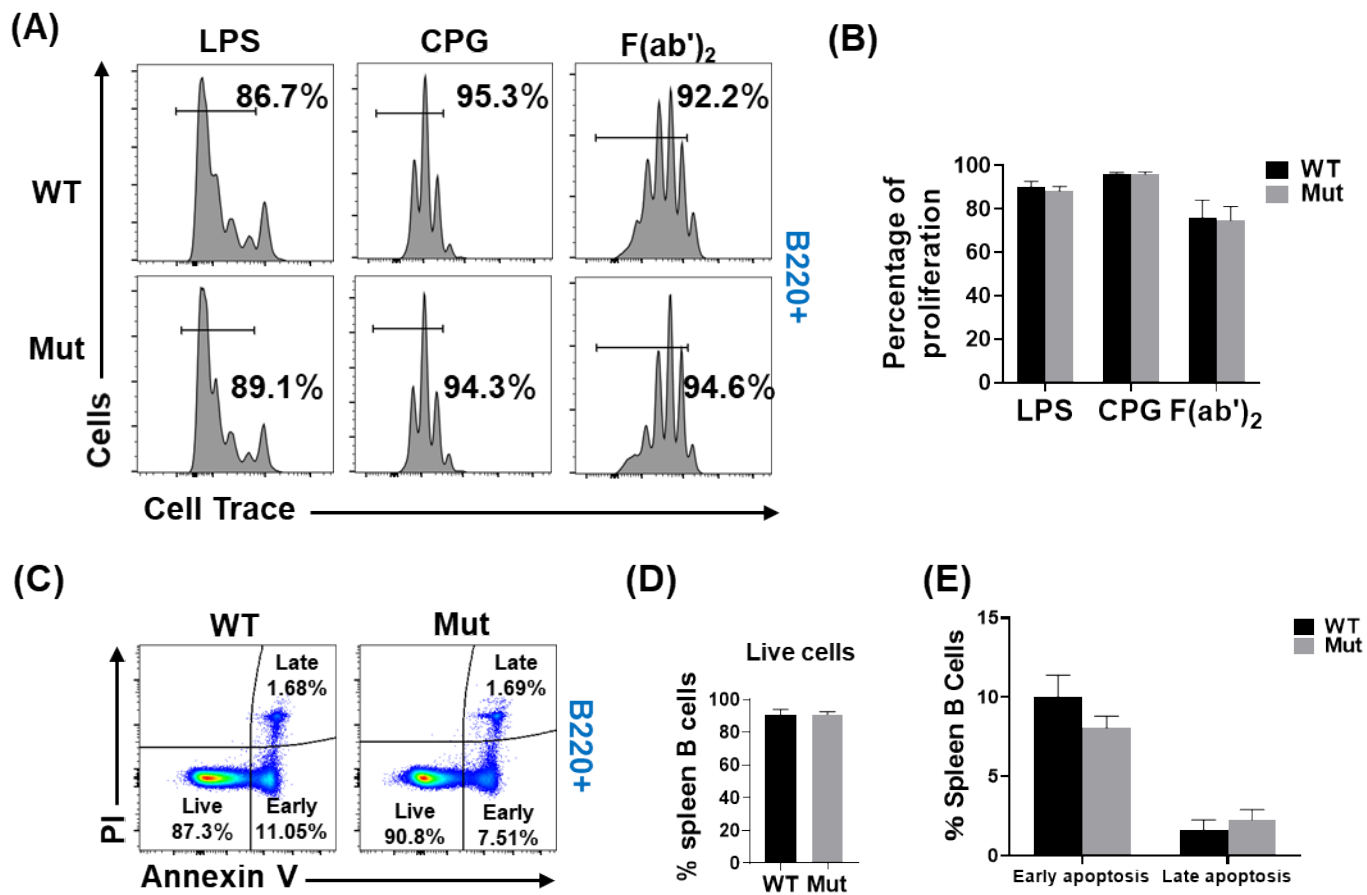

Supplement: Supplementary file 1 — Supplemental Information [file 41419_2024_7180_MOESM1_ESM.pdf]
