# Supplementary material for: DOCK8 gene mutation alters cell subsets, BCR signaling, and cell metabolism in B cells
Source: Cell Death Dis. 2024 Dec 1;15(11):871. doi: 10.1038/s41419-024-07180-w (PMC11608328; doi:10.1038/s41419-024-07180-w)
Supplement: Supplementary file 2 — CDDIS-24-3717R-original data-WB [file 41419_2024_7180_MOESM2_ESM.pdf]

CDDIS-24-3717R-original data-WB

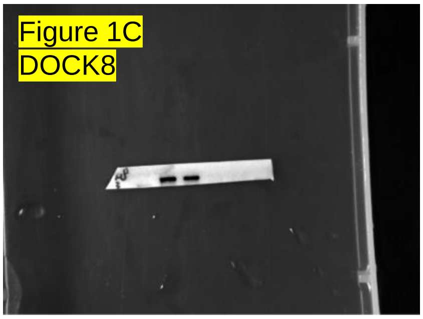
Figure 1C
DOCK8

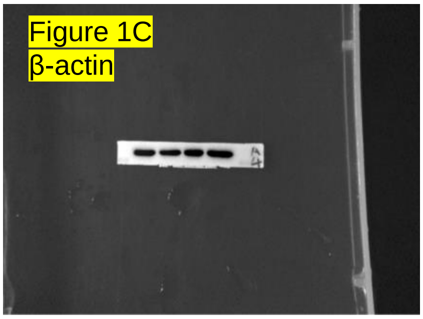
Figure 1C
β-actin

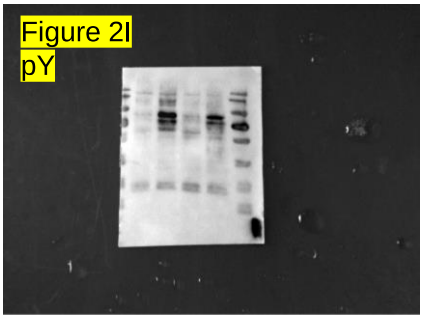
Figure 2I
pY

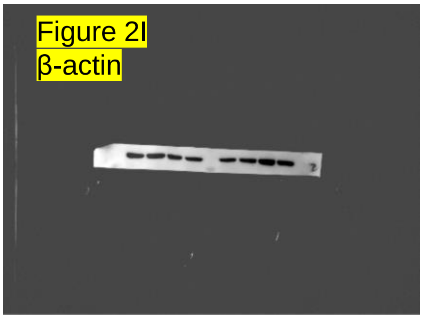
Figure 2I
β-actin

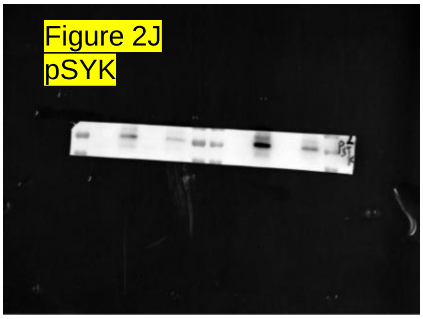
Figure 2J
pSYK

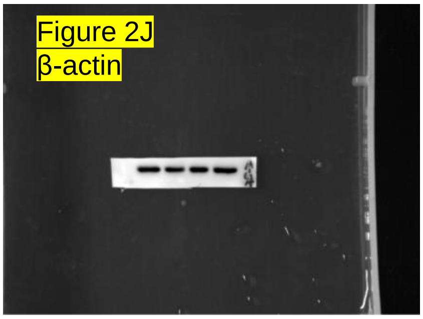
Figure 2J
β-actin

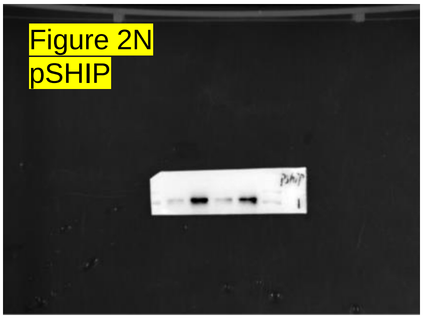
Figure 2N
pSHIP

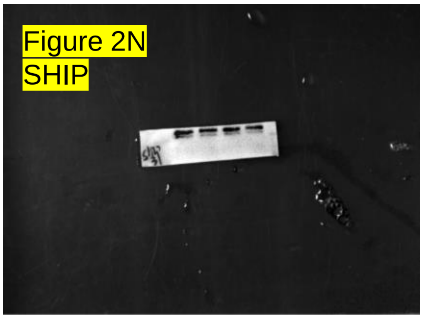
Figure 2N
SHIP

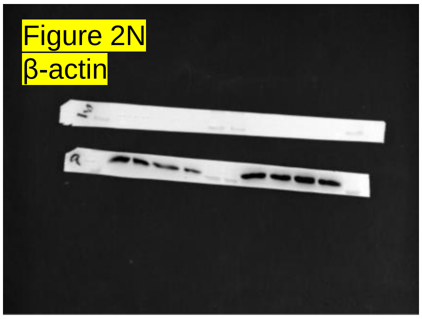
Figure 2N
β-actin

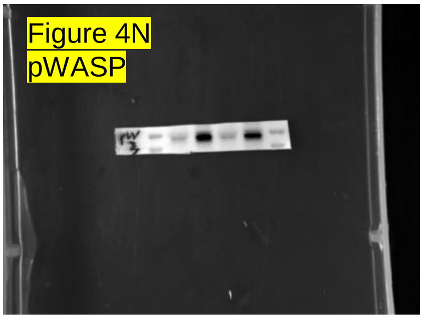
Figure 4N
pWASP

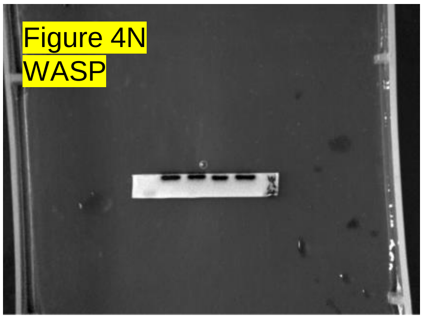
Figure 4N
WASP

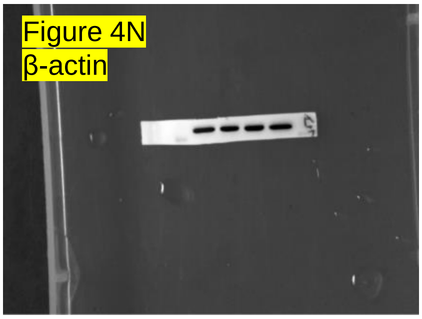
Figure 4N
β-actin

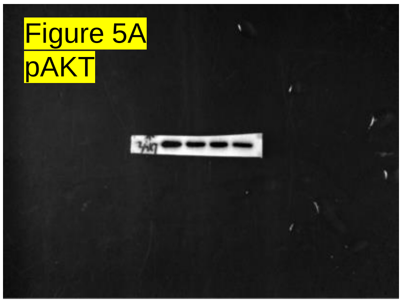
Figure 5A
pAKT

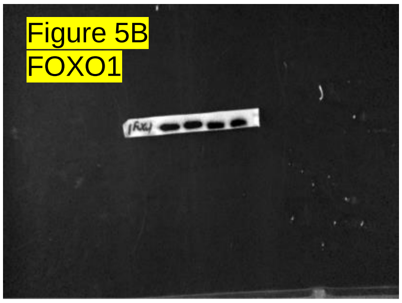
Figure 5B
FOXO1

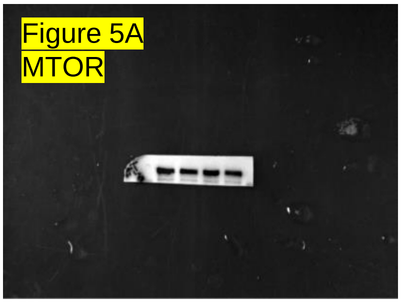
Figure 5A
MTOR

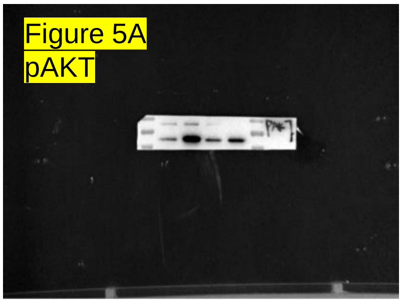
Figure 5A
pAKT

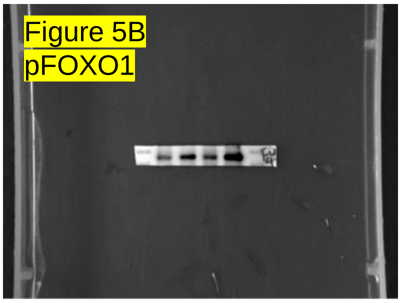
Figure 5B
pFOXO1

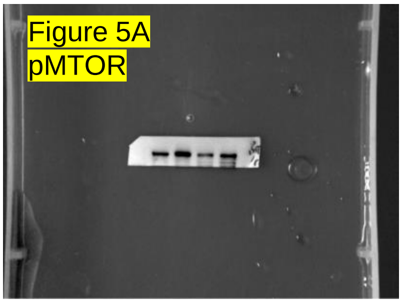
Figure 5A
pMTOR

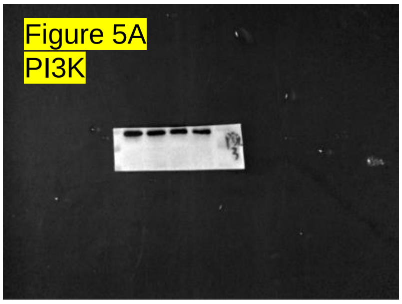
Figure 5A
PI3K

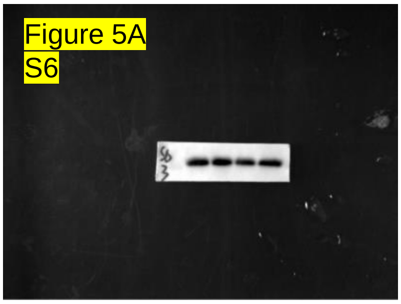
Figure 5A
S6

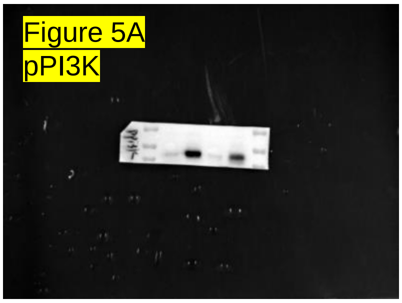
Figure 5A
pPI3K

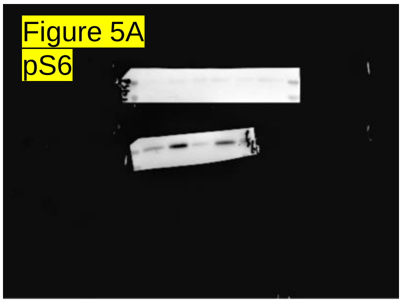
Figure 5A
pS6

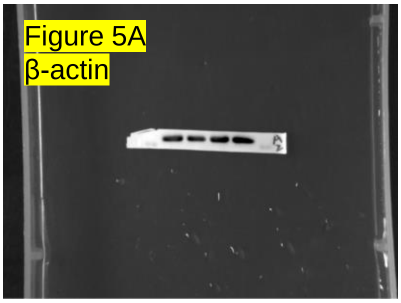
Figure 5A
β-actin

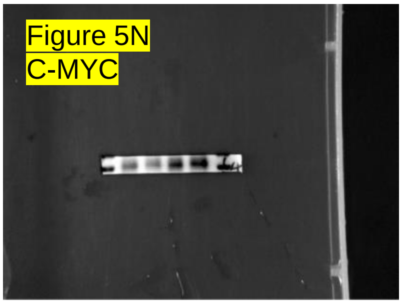
Figure 5N
C-MYC

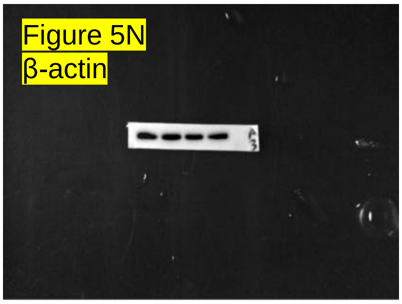
Figure 5N
β-actin

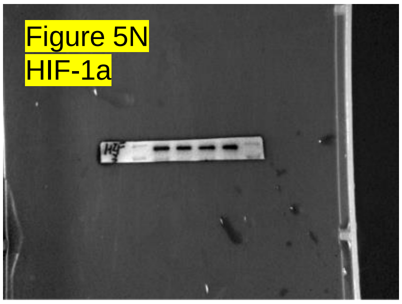
Figure 5N
HIF-1a

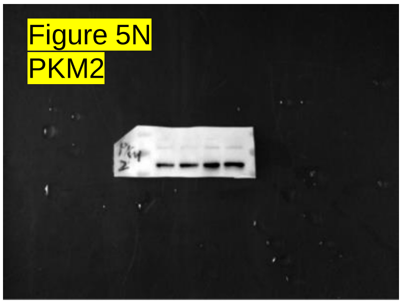
Figure 5N
PKM2

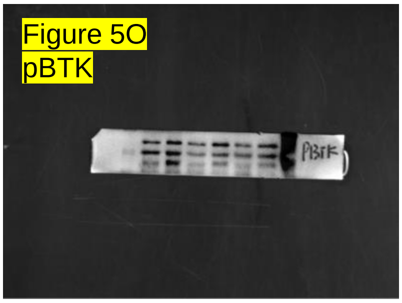
Figure 5O
pBTK

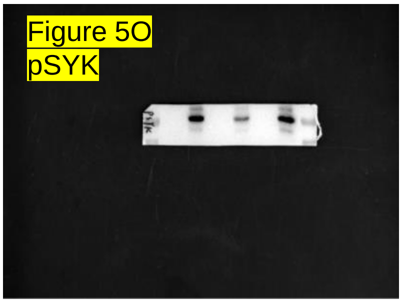
Figure 5O
pSYK

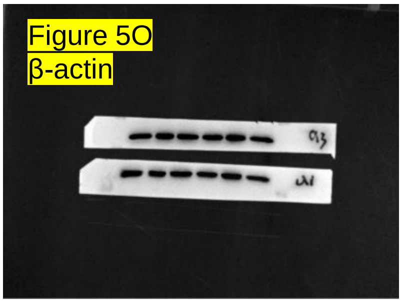
Figure 5O
β-actin
